# De-labelling penicillin allergy in acutely hospitalized patients: a pilot study

**DOI:** 10.1186/s12879-021-06794-1

**Published:** 2021-10-20

**Authors:** Linde Steenvoorden, Erik Oeglaend Bjoernestad, Thor-Agne Kvesetmoen, Anne Kristine Gulsvik

**Affiliations:** 1 Department of Medicine, Diakonhjemmet Hospital, Postboks 23 Vinderen, 0319 Oslo, Norway; 2 Department of Hematology, Oslo University Hospital, Oslo, Norway

**Keywords:** Penicillin allergy, Antibiotic stewardship, Direct oral challenge, De-labelling

## Abstract

**Background:**

Penicillin allergy prevalence is internationally reported to be around 10%. However, the majority of patients who report a penicillin allergy do not have a clinically significant hypersensitivity. Few patients undergo evaluation, which leads to overuse of broad-spectrum antibiotics. The objective of this study was to monitor prevalence and implement screening and testing of hospitalized patients.

**Methods:**

All patients admitted to the medical department in a local hospital in Oslo, Norway, with a self-reported penicillin allergy were screened using an interview algorithm to categorize the reported allergy as high-risk or low-risk. Patients with a history of low-risk allergy underwent a direct graded oral amoxicillin challenge to verify absence of a true IgE-type allergy.

**Results:**

257 of 5529 inpatients (4.6%) reported a penicillin allergy. 191 (74%) of these patients underwent screening, of which 86 (45%) had an allergy categorized as low-risk. 54 (63%) of the low-risk patients consented to an oral test. 98% of these did not have an immediate reaction to the amoxicillin challenge, and their penicillin allergy label could thus be removed. 42% of the patients under treatment with antibiotics during inclusion could switch to treatment with penicillins immediately after testing, in line with the national recommendations for antibiotic use.

**Conclusions:**

The prevalence of self-reported penicillin allergy was lower in this Norwegian population, than reported in other studies. Screening and testing of hospitalized patients with self-reported penicillin allergy is a feasible and easy measure to de-label a large proportion of patients, resulting in immediate clinical and environmental benefit. Our findings suggest that non-allergist physicians can safely undertake clinically impactful allergy evaluations.

## Background

Penicillin allergy is the most commonly reported drug allergy, and the prevalence ranges from 5% in the general population to up to 15% in hospitalized patients [1, 2]. Significant IgE-mediated- or serious non-IgE mediated penicillin hypersensitivity occurs only in a minority of patients, however. Most penicillin allergy reports describe benign cutanous or even unknown reactions [3]. When excluding subjects with a severe type of allergic reaction, 95% of patients tolerate penicillin [2]. Unfortunately, very few patients with reported penicilin allergy undergo any form of evaluation.

Reported penicillin allergies lead to an increased use of broad-spectrum antibiotics with more side effects [4], higher risk of infections with multi-resistant bacteria, including VRE and MRSA [5], and longer hospital stays [1]. Disproving an inaccurate penicillin allergy diagnosis is therefore beneficial for patients, and society benefits from a lower level of antibiotic resistance and lower healthcare costs [6].

Penicillin allergy evaluation in Norway is traditionally carried out in an outpatient allergist clinic as a three-step procedure: (1) in vitro tests of IgE, (2) skin testing and finally (3) an oral challenge. Skin testing consists of several epi- and intradermal injections and can be painful, expensive and time consuming [2, 7]. Furthermore, skin testing yields a large proportion of false positives [8].

An oral challenge is considered to be the gold standard for penicillin allergy evaluation [10]. Several studies have shown that it is safe to test low-risk patients with a direct oral challenge without preceding blood- or skin testing. In these studies, approximately 95% had no immediate reaction [7–11]. Absence of an immediate reaction excludes an IgE-mediated allergy, and future use of penicillin is considered safe [2]. The study population in these studies were mostly patients referred to an allergy clinic [7–9, 12], and young, healthy marine recruits [10]. Previous studies have expressed the need for more testing in non-healthy populations [13] and research done by non-allergists [14].

The aim of this study was to investigate the prevalence of self-reported penicillin allergy in a Norwegian hospitalized patient population, and to study the safety and efficacy of a direct oral challenge in the proportion of these patients with a screened, low pretest probability for a severe allergic reaction. Our hypothesis was that penicillin allergy is over-reported in hospital records and that a direct oral challenge is an easy, cost-effective low-threshold method to rule out the penicillin allergy label for a significant amount of patients during their hospital stays.

## Methods

This study is a prospective interventional study carried out in the Department of internal medicine at Diakonhjemmet hospital in Oslo, Norway. Patients were included during two different time periods, 5 months in 2019 and 3 months in 2020, due to availability of project resources and personnel. All patients admitted to the department of internal medicine were screened by the authors using an electronic patient record system. Patients who self-reported or had a previously documented penicillin allergy were interviewed and assessed for eligibility. Patients reporting reactions defined as “low-risk” were included. Informed consent was obtained from all study participants. The study was approved by the Regional Committee for Ethics in Medical Research (reference nr 2018/1316) and by Diakonhjemmet Hospital Institutional Review Board. All methods were performed in accordance with the relevant guidelines and regulations.

### Definition of ‘low-risk penicillin allergy’

Our definition is adapted from a protocol written by Kuruvilla and Thomas [15]: a low-risk patient has a history of either (a) a benign rash, (b) symptoms unlikely to be of allergic aetiology (e.g. headache, mild GI-symptoms) or (c) no recollection of the reaction. Exclusion criteria were a) prior penicillin-elicited IgE-mediated reactions (e.g. urticaria, angioedema), (b) severe, non-IgE-mediated reactions (e.g. Stevens–Johnsons syndrome, nephritis, hepatitis or other organ involvement), (c) ongoing critical illness (e.g. sepsis with organ failure, decompensated heart failure), (d) recent reaction to penicillin (less than 1 year ago), or (e) severe anaphylaxis of any cause during the last 4 weeks before inclusion. Baseline data were collected from the medical history records, and the participants were interviewed about their index reaction to penicillin.

### Direct graded oral amoxicillin challenge

We chose to test with amoxicillin in the oral provocation test to be able to compare our results to other international studies [8–10, 12, 16]. All tests were performed by one of the authors, all non-allergist medical doctors (LS is a resident in internal medicine, EOB is a resident in hematology, TK is a resident in gastroenterology, AKG is a consultant geriatrician). The patients were tested at the hospital ward they were admitted to. To ensure maximum safety, an intravenous catheter was placed in case of an adverse event that would require intravenous fluid therapy, and an emergency kit with adrenalin was present during the observation period. The patient’s vital signs were measured for reference before testing. After oral administration of the first dose of 75 mg of amoxicillin mixture, the patient was observed for 30 min. When there was no reaction and vital parameters remained stable, the patient received the second dose, 250 mg of amoxicillin mixture. After another 30 min of observation and a third measurement of vital signs, the test was completed. If a patient showed no reaction, an immediate penicillin allergy was disproven. The patient, doctor responsible for in-hospital treatment and general practitioner were informed, and patients were asked to report any late adverse reactions by telephone.

The primary endpoint was the proportion of inpatients who could safely be de-labelled from a reported penicillin allergy. Secondary endpoints included the prevalence of penicillin allergy in the hospitalized patient population, and the practicability of testing inpatients using this method. The data were analysed using Microsoft Office Excel 2019. The study was approved by the Regional Committee for Ethics in Medical Research and by Diakonhjemmet Hospital Institutional Review Board.

## Results

**Fig. 1:**
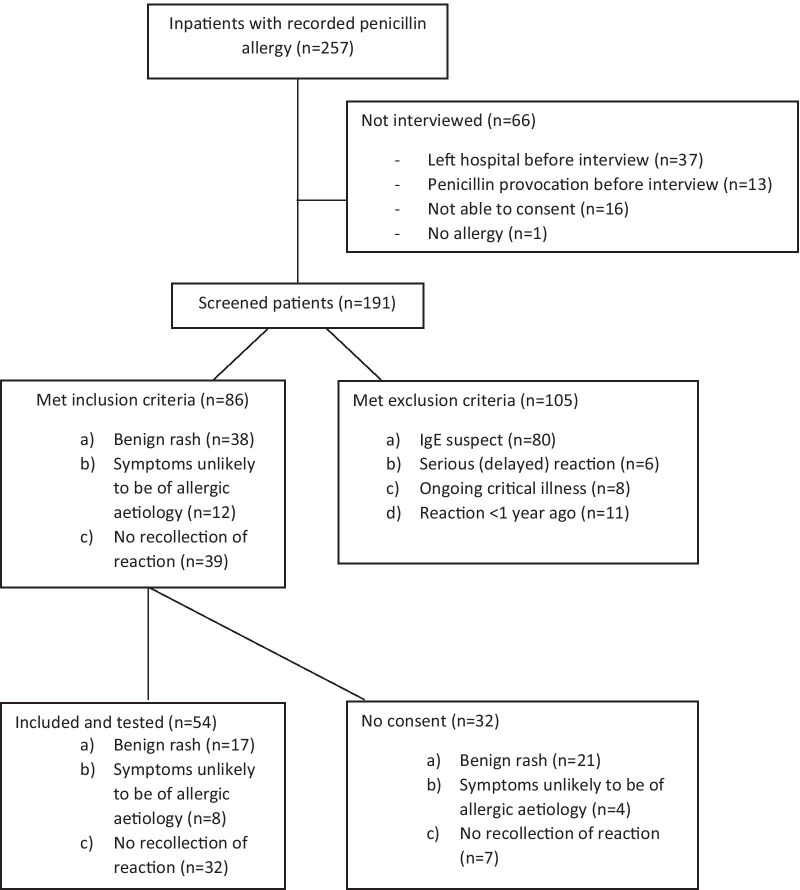
Flowchart of inpatients in the medical department with penicillin allergy reported in their hospital admission records

During the two study periods, 5529 unique patients were admitted to the medical department. 257 of these (4.6%) reported a penicillin allergy, and 47% of these patients were female. The algorithm of inclusion is presented in Fig. 1.

86 (45%) of screened patients met inclusion criteria for testing, of which 54 (63%) were included and tested. 32 patients declined because of anxiety or because they felt too sick or frail to participate.

In addition to the 54 patients included at the medical department, three additional patients were included, from the department of surgery, the emergency department and the geriatric outpatient clinic, respectively. In total, 57 patients underwent the oral provocation test.

Baseline data for the 257 patients with recorded penicillin allergy upon hospital admission are presented in Table 1.

**Table 1:** Baseline characteristics of 257 patients with recorded penicillin allergy at admission

	Eligible patients (meeting inclusion criteria) (n = 89)			Missing patients (not screened by interview) (n = 66)		Excluded patients (n = 105)	
	Consenting and performed oral provocation test (n = 54 + 3 = 57)	Declined oral provocation test (n = 32)	p-value		p-value		p-value^c^
Female N(%)	33 (57)	24 (71)	0.11^a^	42 (63)	0.59^a^	80 (77)	0.02^a^
Age (years) Mean(SD)	68.4 (18.6)	74.7 (17.2)	0.07^b^	70.2 (16.4)	0.30^b^	69.2 (18.8)	0.40^b^

^a^Student’s *t*-test

^b^Chi-squared test

^c^Compared to the consenting patients

The eligible patients who declined to participate in the oral provocation test seemed to be slightly older than those in the group that consented, but these findings did not reach statistical significance, possibly due to lack of power (Table 1).

The eligible patients combined (consenting and declining) (n = 89), had a mean age (SD) of 70.7 (18.3) years, which did not differ from the patients who met the exclusion criteria (n = 105), aged 69.2 (18.8) years (p = 0.29).

The age of the patients in the exclusion group was comparable to the age of the patients in the consenting inclusion group (p = 0.40), but the proportion of women in the exclusion group was significantly higher (p = 0.02). The missing patients did not differ in their baseline data from the consenting patients.

In 80 of the 191 screened patients (42%), an IgE mediated allergy could not be ruled out. 22 (28%) had experienced an urticarial rash, 16 (20%) had a severe anaphylactic reaction, 21 (26%) angioedema and 18 (22%) had experienced difficulties in breathing. Eleven (11%) patients had experienced the allergic index reaction less than 1 year prior to the study screening.

Fifty-six of the 57 patients who were tested did not have an immediate allergic reaction from exposure to amoxicillin, and their penicillin allergy label was thus removed. One of the admitted patients had a worsening of ongoing bronchial obstruction symptoms after the second dose of the oral challenge. Vital signs remained stable, and the patient reported subjective improvement after ipratropium bromide-salbutamol inhalation. His record of penicillin allergy was not indicating any IgE-type reaction, but due to this incident, he could not be de-labelled. One of the other tested patients reported a mild maculopapular rash 2 days after the oral challenge. There was no need for symptomatic treatment.

Twenty-six of the 57 patients who were tested with the oral provocation were under ongoing antibiotic treatment during inclusion. The doctors responsible for in-hospital treatment of these patients were informed. In 11 (42%) of the 26 patients the treating doctor decided it was possible to narrow their ongoing treatment to penicillin immediately after testing. Seven patients were switched to penicillin G/V, the others were changed to either Ampicillin, Amoxicillin or Cloxacillin.

## Discussion

The prevalence of a self-reported penicillin allergy in this Norwegian population was lower than in most previous studies. A true IgE-type allergy was unlikely in 45% of the self-reported penicillin allergic patients, due to an interview algorithm. These patients were offered a direct oral penicillin challenge, of whom 98% of the tested patients had no adverse events and could be de-labeled from penicillin allergy in their hospital records.

Penicillin allergy de-labeling gave immediate clinical and environmental benefit as the antibiotic treatment regimen could be narrowed from a non-penicillin to a penicillin if the patient suffered from a penicillin-sensitive infection when indicated by the Norwegian national guideline of antibiotic use.

The prevalence of penicillin allergy in the hospital records in our medical department inpatient population was 4.6%. This is lower than expected, as the prevalence of penicillin allergy is commonly reported to be around 10% [9, 12, 16, 17]. Other studies have reported penicillin allergy prevalence ranging from 5.9% in the UK general population [18] to 15% in hospitalized patients [1]. The variable findings may in part be due to the differences in age and health status in the different study populations. Another possible reason for the low prevalence finding in our study might be that antibiotic use in Norway is among the lowest in the world, thus leading to a lower prevalence of antibiotic-associated adverse drug reactions [19]. A study published in 2006 with a study population of admitted patients in a hospital in Denmark, a country with similar antibiotic use to Norway, reports a prevalence of 5%, which is in line with our study [20]. They state that penicillin G/V is used more often in Denmark than in other European countries, which tend to use more broad-spectrum penicillins. Broad-spectrum penicillins are associated with a higher risk for adverse drug reactions compared to penicillin G&V [20]. To our knowledge, no previous studies have been conducted in a random sample of acutely admitted patients in a Norwegian medical department, and the present study adds to current knowledge on penicillin allergy over-reporting in hospital records.

Our study also shows that a direct oral amoxicillin challenge is an easy and a safe way to rule out penicillin allergy in acutely hospitalized patients, when carefully selected. 98% of the tested patients had no immediate reaction. Previous comparable studies with varying study populations have reported 94–98% tolerance to the oral challenge [7–9, 11, 16, 17], and none of them have reported severe immune-mediated reactions due to the provocation test, when inclusion criteria were met.

Fortysix percent of the tested patients were under treatment with antibiotics at the time of testing. For 42% of these patients, their in-hospital antibiotic treatment was changed from a non-penicillin to a penicillin, which shows an immediate effect of inpatient penicillin allergy de-labelling. Patients with a negative allergy test who did not change treatment to a narrow spectrum penicillin were either not currently admitted because of an infection, or they suffered from infections/bacteria not susceptible to penicillin treatment (either based on microbiological findings or based on the empirical Norwegian national guidelines for antibiotic use). In a comparable study, 56% percent of inpatients used beta-lactam antibiotics subsequent to testing [11].

A downside of testing acutely hospitalized patients is that 36% of the eligible inpatients did not consent to testing because of anxiety or because they felt too sick or frail to participate. In comparable studies with inpatients, 37% and 48% did not consent to testing [16, 17]. The non-consenting patients did not differ from the consenting patients in their baseline characteristics (Table 1). There are, however, other differences. Sixtysix percent of the non-consenting patients reported to have had a benign rash as their index reaction to penicillin, as opposed to only 30% of included patients. In addition, as many as 56% of included patients had no recollection of their reaction; for patients that did not consent, this was 22%. This indicates that patients who remember their index reaction, or had a benign but bothersome rash, and thus may have had a more serious reaction, are less likely to consent to the oral challenge, which may represent a bias. A study by Siew et al. has indeed shown that patients who recall their index reaction to beta-lactam antibiotics are associated with increased probability of a true allergy [21].

Our study followed quite strict exclusion criteria compared to similar studies [9, 16]—we excluded not only life-threatening reactions, but all patients with a possible IgE-mediated reaction. Only 45% of the screened patients were identified as low-risk. Roughly half (47%) of all admitted patients were female; of the screened patients with a reported penicillin allergy, 69% were female. The even higher proportion of women in the exclusion group might indicate that women are more prone to penicillin allergy than men, but it may also mean that women have had more previous antibiotic exposure, or that they recall and report previous allergic symptoms to a higher extent than men. Female sex as a risk factor for penicillin allergy, and adverse drug reactions in general, has been described in previous studies [22] 23. Among the excluded patients, there might have been some who did not have a true IgE type allergy; thus, our strict screening algorithm might have contributed to exaggerating rather than deflating the estimated number of patients with suspected IgE type allergy.

It might seem excessive to test low-risk patients, and in the future it might be concluded that it is not necessary to expose these patients to testing entirely. However, in the current situation these patients continue to report they are allergic to penicillin. Compared to the costs of using alternative antibiotics, which are associated with longer hospitalisations, higher risk of adverse drug reactions and increasingly resistant bacteria, the benefits of oral challenges outweigh the costs.

Our study has several limitations. Firstly, the study was small and carried out in a single local hospital in Oslo, Norway, with a predominantly ethnic Norwegian population of high socioeconomic status.

Secondly, we only tested to exclude immediate IgE type reactions. However, studies that have tested patients with a multiple-day course of penicillin report a prevalence of mild delayed reactions close to the baseline incidence of the general population, and testing for a longer period of time causes unnecessary exposure to antibiotics [2, 7]. Thirdly, enteral exposures have a reported lower risk of anaphylaxis than intravenous exposure [2]. It might be possible that the rate of allergic reactions would have been higher (and more accurate) if patients were tested with intravenous penicillin rather than through enteral admission. Fourthly, data were collected in two separate periods by three different medical doctors. Fifthly, 42% of screened patients were excluded because an IgE-mediated reaction could not be ruled out. This includes patients who self-reported urticaria, relying on their ability to distinguish urticaria from other rashes. This could contribute to either under- or over-estimation of IgE-mediated reactions. Furthermore, we did not compare findings to placebo, in contrast to another study [9], but since there were no patients reporting any subjective reactions during testing, there does not seem to be an obvious need for placebo comparison, and we do not expect these factors to influence our findings in a significant way.

Our protocol definition of low-risk allergy [15] included symptoms of intolerance (e.g. headache, mild GI-symptoms) and consequently these patients where challenged. These patients have a very low probability of having penicillin hypersensitivity, and thus comparable studies using other protocols have de-labelled this group of patients through medical history alone, not offering oral challenges [24]. One could argue that offering these patients oral challenges would yield a falsely high rate of negative tests. However our results are similar to the above mentioned study [24] suggesting that the rate of negative challenges is not influenced by our selection of patients. For patients with no medical understanding of allergic reactions, undergoing an oral challenge can feel reassuring, and thereby contribute to refraining from reporting a penicillin allergy in the future.

As oral penicillin challenges in low-risk patients have been demonstrated to be safe, future studies might even look into the possibility of by-passing the oral penicillin challenge and de-label penicillin allergy solely based on the specificity of the inclusion criteria. Further research is warranted to develop a standardized algorithm to identify low-risk patients, as previous studies have used different methods.

## Conclusions

The prevalence of self reported penicillin allergy was low in this Norwegian hospitalized population, and lower than in most previous studies. Furthermore, a true IgE-type allergy was unlikely in 45% of the patients reporting on penicillin allergy. A direct oral penicillin challenge was a safe and efficient method for de-labelling penicillin allergy in these patients during their hospital stay. The oral penicillin challenge gave immediate clinical and environmental benefit as the antibiotic treatment regimen could be narrowed from a non-penicillin to a penicillin, if the patient suffered from a penicillin-sensitive infection when indicated by the Norwegian national guideline of antibiotic use. Our findings suggest that non-allergist physicians can safely undertake clinically impactful allergy evaluations.

## Data Availability

The datasets used and/or analysed during the current study are available from the corresponding author on reasonable request.
